# Unlearning prohibition: the de-implementation of HIV breastfeeding restrictions in the United States

**DOI:** 10.3389/frph.2026.1869376

**Published:** 2026-07-22

**Authors:** Paige M. Anderson, Molly Bachmann, Marielle S. Gross

**Affiliations:** 1 Department of Obstetrics and Gynecology, School of Medicine, University of North Carolina at Chapel Hill, Chapel Hill, NC, United States; 2 Department of Health Management and Policy, School of Public Health, University of Michigan, Ann Arbor, MI, United States; 3 Center for Health Justice & Bioethics, Lewis Katz School of Medicine, Temple University, Philadelphia, PA, United States

**Keywords:** bioethics, implementation science, infant feeding, obstetrics, patient autonomy, perinatal HIV, reproductive justice, stigma

## Abstract

In January 2023, U.S. perinatal HIV guidelines shifted to support shared decision-making in infant feeding, ending categorical prohibition of breastfeeding for people living with HIV (PLHIV). This change did not reflect new safety evidence, low transmission risk and near-normal life expectancy with antiretroviral therapy has been established for over a decade, but the convergence of evidence with sustained ethical critique of the harms of prohibition. Three years later, uptake remains inconsistent: clinicians report uncertainty, and patient experiences suggest limited change in counseling or practice. We argue that eliminating prohibition of breastfeeding for PLHIV is fundamentally a de-implementation challenge. It calls for deconstruction of a practice that functioned not only as clinical guidance but as moral certainty, reinforced by legal and institutional structures. Standard implementation strategies were never designed for this task. Effective change requires deliberate unlearning of outdated assumptions at the individual and institutional level, healing between clinicians and communities whose trust was broken, and active dismantling of the legal, structural, and cultural infrastructure that encoded prohibition. We outline priorities, including structured unlearning activities, institutional reform, equity-centered training, privacy-protective research infrastructure, and advocacy for repeal of HIV criminalization statutes. The goal is to replace a paternalistic, physician-centered fiction of zero-risk with an allyship model where women’s and infants’ interests are maximally advanced in collaboration with their physicians, systems, and communities.

## 1 Introduction

In January 2023, U.S. perinatal HIV guidelines were revised to support shared decision-making in infant feeding, affirming that people living with HIV (PLHIV) who meet clinical criteria may be supported in breastfeeding (1). This marked a departure from prior guidance that treated breastfeeding as contraindicated for PLHIV. The shift reflects alignment with long-standing scientific data and sustained ethical critique documenting harms of prohibition and conflicts with autonomy, beneficence, and reproductive justice (2–8).

A reproductive justice framework clarifies these harms: beyond limiting choice, prohibition denied a meaningful dimension of parenting to populations already affected by stigma and structural inequity (4). In the U.S., HIV disproportionately affects Black individuals, immigrants, and those with low socioeconomic status, groups that also encounter systemic barriers to breastfeeding (9–12). Implementation of the new guidelines therefore requires more than permission to breastfeed; it requires dismantling the clinical and institutional frameworks that made prohibition permissible.

Implementation has been uneven in the three years since U.S. guidelines changed. Surveys of clinicians document variability in uptake of the revised guidelines, inconsistent application of eligibility and monitoring practices, and persistent provider uncertainty about the evidence, ethics, and logistics of the new approach (13, 14). Patient reports mirror these findings, describing conflicting messages, limited support, and clinical encounters that are unchanged in tone or practice (15, 16). Together, these findings suggest that the infrastructure of prohibition, including clinical heuristics, EHR defaults, institutional policies, and professional norms, remains largely intact.

Standard implementation approaches, including protocol updates, clinician training, and system redesign, are necessary but insufficient. The revised guideline is fundamentally a de-implementation challenge: removing a deeply rooted institutional practice of categorical prohibition of breastfeeding for PLHIV embedded not only in workflow but in moral reasoning shaped by decades of HIV stigma (17). Effective change requires dismantling those beliefs and their institutional remnants, repairing harm they caused, and ensuring PLHIV receive individualized care free from the moral framework that previously justified prohibition.

### 1.1 A history that explains the present

**Figure 1:**
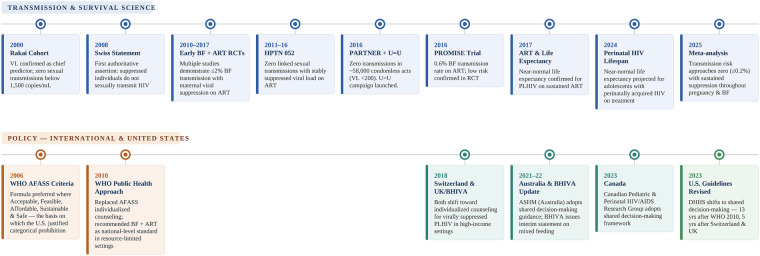
Two-track timeline of HIV transmission science and infant feeding guideline changes. The upper track (Transmission & Survival Science) summarizes key transmission and survival evidence from the Rakai Cohort (2000) through a 2025 meta-analysis showing near-zero transmission with sustained viral suppression. The lower track (Policy: International & United States) summarizes WHO milestones, high-income country shifts to individualized and shared decision-making approaches (Switzerland/UK 2018, Australia 2021–22, Canada 2023), and the 2023 U.S. DHHS revision. The figure illustrates the lag between the accumulation of supportive evidence and U.S. policy change. ART, antiretroviral therapy; AFASS, acceptable, feasible, affordable, sustainable, and safe; ASHM, Australasian Society of HIV, Viral Hepatitis and Sexual Health Medicine; BF, breastfeeding; BHIVA, British HIV Association; DHHS, U.S. Department of Health and Human Services; PLHIV, people living with HIV; U=U, Undetectable=Untransmittable; VL, viral load; WHO, World Health Organization.

The science underlying the 2023 guideline change has existed for well over a decade. By the early 2000s, evidence established that HIV transmission risk correlates with viral load, with negligible risk under suppression (18). By the 2010s, subsequent landmark studies confirmed no linked transmissions with sustained viral suppression, underpinning the U=U (Undetectable=Untransmittable) movement, and multiple studies demonstrated transmission rates below 1% during breastfeeding among women virally suppressed on antiretroviral treatment (ART) (19–24). In 2016, the PROMISE trial further confirmed the low risk of transmission through breastfeeding, demonstrating a 0.6% transmission rate, and a 2025 meta-analysis found that with sustained viral suppression throughout pregnancy and breastfeeding, transmission risk begins to approach zero (0.2% or lower) (25, 26). Beyond reduced transmission rates, health outcomes for PLHIV have greatly improved over time. With consistent ART use, individuals now have life expectancies that are nearly normal, and recent evidence suggests this holds true for those who acquired HIV perinatally and receive proper treatment (27, 28). See Figure 1 for a historical timeline.

This evolving evidence shaped international policy well before the U.S. revised its guidelines. The World Health Organization (WHO) recommended breastfeeding for PLHIV on ART in resource-limited settings in 2010, and several high-income countries later adopted individualized counseling approaches (29–34). By contrast, the U.S. maintained categorical prohibition until 2023, applying the logic of the WHO's 2006 AFASS criteria, that formula was preferred where acceptable, feasible, affordable, sustainable, and safe, as though those conditions were universally met in the U.S. (35).

This pattern of restrictive policy for PLHIV extends beyond infant feeding. The FDA categorically prohibited men who have sex with men from donating blood until 2023, with revision similarly following ethical pressure rather than net-new evidence (36). HIV criminalization statutes in 34 U.S. states enable prosecution of behaviors whose transmission risk is often negligible with ART, many with “bodily fluid” language broad enough to encompass breast milk (37). Globally, at least twelve women have been criminally prosecuted for breastfeeding while living with HIV (38). In the U.S., the threat has operated primarily through child protective services (CPS) involvement. Cox et al. extensively document that CPS referrals have been initiated by clinicians themselves, disproportionately targeting BIPOC families, compounding medical mistrust and creating material barriers to care (39). Such actions in healthcare contribute to the criminalization of HIV status and reinforce moral and ethical frameworks that underpin the prohibition of breastfeeding for PLHIV (3, 36, 37).

This history is not simply a story of policy catching up with science. It is a story of how HIV stigma and fear are so deeply institutionalized in the fabric of American society and medicine that even a fundamental transformation of its clinical reality could not dislodge them. ART transformed a once rapidly fatal, highly transmissible infection into a manageable chronic condition with near-normal life expectancy and negligible transmission risk. That transformation is among the most dramatic in modern medicine. Nevertheless, the overarching approach aimed at eliminating all risk, reflected in the prohibition of breastfeeding for PLHIV, persists. This is not due to ignorance of the available data, but rather stigmatizing perceptions of HIV deeply embedded in our legal and medical institutions (39).

### 1.2 What history produced: the barriers to implementation

The 2023 guideline change represents an important improvement, but it does not automatically undo what decades of prohibition have built. Research suggests prohibition of breastfeeding for PLHIV has caused fracturing of patient-provider relationships, covert breastfeeding without clinical support or safety monitoring, and missed opportunities to support maternal and infant health (40, 41). Over forty years of categorical prohibition have resulted in a research landscape shaped by this restrictive approach. Consequently, critical topics such as optimal viral load monitoring during breastfeeding, the safety of mixed feeding practices, the management of complications, and infant prophylaxis within the U.S. context remain insufficiently studied (13, 14, 42).

What remains of this landscape is informative qualitative data. PLHIV in the U.S. describe experiences of distrust, coercion, threats of CPS involvement, and clinical encounters in which they felt they were perceived as not having their infants' best interests in mind (Table 1) (13–16, 41–44).

**Table 1:** Representative voices: patient and clinician perspectives.

***Patient Perspectives*** Qualitative research with PLHIV in the U.S. reveals how the prohibition and its clinical enforcement damaged trust, denied autonomy, and left patients feeling that the medical system viewed them as threats to their own children rather than partners in their care. *“You do get mad because it's like a violation of your privacy…it's your family and you have someone telling you, ‘you have no choice’. And then there's that background, ‘Well, I might call CPS on you’…You can't trust them.”* (15) *“I want a provider to … know that I love my baby more than they do… Treat me like that… Just assume that I’m making decisions with my baby's best interest in mind.”* (15) *“If I have an undetectable viral load, and I can't transmit the virus, how can I transmit the virus through breastfeeding? Does breastmilk become magically detectable while the rest isn't? Seriously. I’m serious.”* *— Mother living with HIV* (41) *“They should offer. They didn't offer. They didn't ask me if I wanted to breastfeed.”* (43) ***Clinician Perspectives*** Provider responses to the revised guidelines reflect a spectrum, from deeply held protectionist instincts and zero-risk thinking that resist the shift, to cautious engagement, to genuine relief at the change, illustrating both the depth of the unlearning required and the possibility of it. *“I feel the need to protect the infant and think it isn't ethical to put the infant at increased risk.”* (42) *“In our country where formula [is] easily accessible, even one single baby infected via breastfeeding would be ethically incorrect.”* (13) *“In my clinical setting many patients have unreliable continuity of care, or are unreliable when taking medications.”* (13) *“The [new] guidelines were very helpful. I’ve just been so happy about the change in verbiage… to decrease stigmatization.”* (13)

Quotes are drawn from qualitative research with people living with HIV and clinicians in the United States. Numbers in parentheses refer to the corresponding works in reference list that these quotes were sourced from.

These experiences are disproportionately documented among PLHIV from marginalized communities already at greater risk of discrimination and state surveillance, compounding existing health inequities (39, 45). This inequity has not changed with guideline changes; it is reproduced where root causes are unaddressed, including through the risk of selective or inconsistent counseling under the new guidelines. The same clinical environment that produced this distrust, the blurring of care and surveillance, excluding affected communities from decision-making, and leaving institutional policies and criminalization statutes intact, continues to shape how patients and clinicians meet each other (46).

The heuristics clinicians carry, the defaults still embedded in EHRs and hospital policies, the criminalization statutes that remain on the books, and the memory patients hold of being shamed or threatened, none of these are automatically corrected with guideline updates. Rebuilding begins with deconstruction: dismantling the clinical, institutional, and legal infrastructure that produced these conditions in the first place.

## 2 Discussion: de-implementation & the case for “unlearning”

The 2023 guideline change is a paradigm case for de-implementation: the field is being asked to stop doing something clinicians have been trained, institutionally reinforced, and morally socialized to do for nearly four decades. De-implementation, the deliberate removal or replacement of an established clinical practice, is conceptually distinct from implementation, and ignoring de-implementation in the era of these new guidelines may result in failure (17). This difference has been described as asymmetry. Adopting a new practice asks clinicians to build something new while de-implementation asks them to dismantle existing structures, which they may have helped construct and are rewarded for maintaining (47). De-implementation also requires replacing a negative rule, “do not breastfeed,” with a more positive and resource-intensive one: individualized counseling, active support, and shared decision-making. Replacement is harder than adoption of a novel practice, because the old rule must be actively suppressed to build the new (48).

The reason de-implementation is distinct lies in how established clinical practices are stored. Helfrich and colleagues apply dual-process cognitive theory to explain why evidence updates so often fail to change clinical behavior; entrenched clinical rules become automatic, fast, cue-triggered responses that do not require deliberate reasoning to execute (49). Evidence engages the slow, reflective system; the heuristic lives in the fast, automatic one. A clinician can consciously accept that breastfeeding transmission risk under viral suppression is below 1% and still reflexively counsel against it at the bedside because the heuristic activates before reflection begins. This is not ignorance or bad faith. It is how deeply learned clinical rules operate. Schema theory expands this; established mental models actively filter contradicting evidence, bending new information to fit the existing framework rather than revising it (50). When a clinical practice has also become a moral conviction, “this is what a responsible clinician does to protect the baby,” the resistance compounds. The cognitive and structural frameworks that sustained the practice must be actively addressed, not assumed to dissolve when a guideline changes (13, 14). Organizational behavior theory adds that clinical prohibitions embed over time at the level of institutional assumptions, no longer consciously held policies but unspoken norms that shape behavior without reflective examination (51, 52). While the dual-process model may oversimplify decision processes, its integration with complementary theoretical frameworks offers a more robust foundation for de-implementation efforts, though real-world applicability requires further validation (53).

De-implementation may be uniquely challenging in perinatal care. Obstetrics is distinctive among medical specialties in invoking a perceived duty to protect a third party, the fetus or newborn, as moral justification for practices that limit patient autonomy (54, 55). However justified, this has resulted in paternalism toward birthing people: replacing the patient's assessment of risks and benefits with the clinician's judgment, a practice reinforced by medicolegal and socioeconomic norms.

For example, continuous electronic fetal monitoring in low-risk labor has persisted at over 85% of U.S. deliveries for more than thirty years despite evidence showing that it does not reduce perinatal adverse outcomes and significantly increases cesarean delivery (56). Likewise, “trial of labor” after cesarean gained uptake in the 1980s and 1990s following guideline endorsement, but rates fell sharply in the early 2000s as liability concerns drove hospital-level institutional bans rather than formal guideline withdrawal, showing how nationally endorsed practices can be effectively disregarded through local institutional and professional pressures when liability fears and underlying incentives are insufficiently addressed (57–62).

HIV and breastfeeding decisions follow and magnify this pattern: moralized prohibition was reinforced by clinical norms and liability fear as well as criminalization statutes, CPS surveillance, and four decades of societal HIV stigma. Although several of these practices have gradually phased out, the slow pace is unacceptable. PLHIV have lost more than a decade to guidelines that the evidence no longer supported.

Unlearning is a central mechanism of de-implementation. Tsang and Zahra define organizational unlearning as the deliberate suspension of an established routine's governing authority, not the erasure of knowledge, but the active suppression of its claim to direct behavior (63). Three depths of unlearning have been described: routine unlearning, the discarding of outdated specifics; wiping, the deliberate clearing of knowledge to make room for new content; and deep unlearning, the fundamental disruption of underlying assumptions, typically accompanied by emotional resistance, identity disequilibrium, and the unsettling of professional self-understanding (64).

The guideline change requires deep unlearning because prohibition of breastfeeding was a moral framing to protect the infant. For a clinician who practiced under this framework in good faith, accepting the new evidence means confronting the possibility that they may have caused harm to the patients and families they were trying to protect. That confrontation is emotionally and professionally demanding. If a system fails to create space for this deep unlearning, the consequences surface as clinical behaviors: reluctance to change, language that updates without practice following, or outright rejections (Table 1).

Given that language carries ethical assumptions, unlearning requires scrutiny of clinical language. While the 2023 guidelines call for “shared decision-making,” this recommendation is frequently operationalized via a harm-reduction framing (65). Harm reduction has its roots in addiction medicine, and while preferable to prohibition, carries an implicit assumption that breastfeeding is a deviant risk that medicine is tolerating rather than a recognized health practice with established benefits for both parent and infant (2, 66). A more precise and ethically accurate framing is risk-informed, autonomy-centered care: one that situates a low but non-zero transmission risk alongside the real and documented benefits of breastfeeding, affirms all feeding options as legitimate health practices rather than a preferred (formula) and a tolerated one (breastfeeding), and centers the patient's individual circumstances and values in the process. This reframing is consistent with Cox and colleagues' Informed Free Choice framework, which centers patient agency and situates counseling within the relational and structural conditions that make genuine choice possible (39).

Deep unlearning also requires deliberate structured activities that create conditions for cognitive and relational change in a psychologically safe environment. At the individual level, this could mean case-based reflection sessions that walk clinicians through the evidence-policy gap: not just presenting new data, but explicitly examining how the prior framework was formed, what it assumed, and what it cost. These reflection exercises should also include personal reflection: reconciling the distance between stated values and actual practice in a way that motivates and heals. These activities should not happen in clinical silos but should involve communities to promote change and trust. This can be accomplished by events that bring clinicians and patients into genuine dialogue, such as case conferences, community advisory feedback sessions, and narrative medicine approaches. Unlearning must be paired with replacement; clinicians who have deconstructed the old framework need concrete tools to practice the new one. The goal is not just a change in clinician recommendation, but the development of genuine fluency in counseling for informed, supported choice, and a clinical environment structured to make that path feasible.

Beyond unlearning, literature identifies several other conditions necessary for de-implementation to succeed at scale (39, 48, 67). Community engagement is not only a key condition for unlearning, but also for shaping how institutions should execute these new guidelines. Cox and colleagues have developed the most complete framework for what is required in this context: genuine partnership with PLHIV as co-designers of care, not just consultants to clinician-led processes, in order to build shared spaces for bidirectional reckoning and healing (39). Peer champions will be needed: respected colleagues who model the new approach publicly, creating social permission to change (67). And the structural cues that continue to trigger the old heuristic, including EHR defaults, order sets, and documentation templates that still encode formula as standard, must be actively removed, because as long as they remain, the clinical environment will keep defaulting behavior back to prohibition (48).

### 2.1 Rebuilding: priorities after de-implementation

While distinguishing de-implementation from implementation is important, it does not negate the need for implementation efforts. Indeed, the literature shows that they are most effective when paired together (47). Implementation efforts will require explicit priority setting as well.

Equity must be embedded within program implementation, not assumed. Without standardized equity-audited counseling processes, access to breastfeeding support will follow the same implicit judgments that shaped selective patient surveillance under prohibition. Eligibility assessment and monitoring expectations should be transparent and consistently applied, and disparities in outcomes and clinician recommendations should be routinely evaluated (1, 39). Clinician education and institutional protocols should make it explicit that breastfeeding decisions alone are not grounds for CPS involvement. Logistical burdens such as monitoring costs, transportation, inflexible work schedules, and limited lactation infrastructure must also be addressed. Equity-conscious implementation could include home-based laboratory monitoring, tele-lactation, coordination with WIC and home visiting programs, and expanded access to long-acting injectable ART that could address adherence concerns, though their safety in breastfeeding remains understudied and closing that gap is itself a priority (68).

Trust repair will require visible institutional action. The relational work of healing must be matched with interdisciplinary care teams that include lactation consultants trained in HIV, clear confidentiality protections, and referral pathways that do not require patients to re-navigate the same institutions where they were previously threatened (39). Clinicians and professional organizations should also advocate for repeal of HIV criminalization statutes. For as long as there is a perceived connection between care and potential criminalization, the clinical relationship cannot be whole.

Closing the research gap that prohibition created requires privacy-protective data infrastructure that allows patients to share breastfeeding experiences without fear of legal or social consequences. Community-partnered research addressing questions that prohibition left unanswered, such as optimal monitoring frequency, mixed feeding safety, and complication management, can help shape institutional policy and support patients who cannot meet rigid protocol requirements by providing more safety data.

Finally, the de-implementation and unlearning described here are relevant beyond perinatal HIV care. Categorical prohibition is a recurring posture across perinatal medicine. Two examples worth naming are infant sleep and home birth. The medical establishment's strict prohibitive stance on co-sleeping practices and home birth fails to control people's behavior and drives those who wish to engage in these practices outside of clinical relationships, producing net harm rather than safeguards (69, 70). Although the substantive contexts differ from HIV in their moral, legal, and epidemiological stakes and are not directly analogous, the intellectual move required is similar: moving away from categorical thinking and toward a clinical approach that supports patient agency, accommodates natural biological processes, and minimizes harms broadly construed. Countries including the U.K., Norway, and the Netherlands have achieved comparable and better outcomes through this type of model, demonstrating that this shift is both possible and overdue (69).

## 3 Conclusion

The 2023 guideline revision represents a meaningful shift, but policy change alone does not produce practice change. Practice change requires prioritizing de-implementation: unlearning outdated assumptions, repairing trust, and dismantling the institutional remnants of prohibition. Only then can the field turn fully to closing structural, research, and innovation gaps in optimal perinatal HIV care. The goal is not simply to allow breastfeeding for PLHIV, but to ensure that care delivery, and the systems that shape it, reflect autonomy, equity, and reproductive justice.

## Data Availability

The original contributions presented in the study are included in the article/Supplementary Material, further inquiries can be directed to the corresponding author.
